# Linezolid Toxicity and Mitochondrial Susceptibility: A Novel Neurological Complication in a Lebanese Patient

**DOI:** 10.3389/fphar.2016.00325

**Published:** 2016-09-20

**Authors:** Ossama K. Abou Hassan, Mohamad Karnib, Riyad El-Khoury, Georges Nemer, Mamdouha Ahdab-Barmada, Pierre BouKhalil

**Affiliations:** ^1^Department of Internal Medicine, American University of Beirut Medical Center, American University of Beirut, BeirutLebanon; ^2^MMA-Neuromuscular Diagnostic Lab, Department of Pathology and Lab Medicine, American University of Beirut Medical Center, American University of Beirut, BeirutLebanon; ^3^Department of Biochemistry and Molecular Genetics, American University of Beirut, BeirutLebanon

**Keywords:** linezolid, toxicity, globus pallidus, mitochondria, J1 haplogroup

## Abstract

The recent rise in the use of linezolid to treat a variety of resistant pathogens has uncovered many side effects. Some patients develop lactic acidosis, myelosuppression, optic or peripheral neuropathies, and myopathies. We evaluated an elderly patient who presented to the Emergency Room with linezolid toxicity and a novel neurologic complication characterized by bilateral globi pallidi necrosis. Mitochondrial ribosome inhibition was described to be the predisposing factor. The patient belongs to the mitochondrial J1 haplotype known to be associated with side effects of the drug. We recommend based on the molecular profile of the illness pretreatment considerations and complication management.

## Introduction

A 74 years old Caucasian man presented to the Emergency Department because of altered level of consciousness. He had undergone a recent right knee prosthesis implantation and had been on antibiotic therapy for 5 weeks for prosthesis infection. Initially, he received 14 days of intravenous empiric antibiotic therapy and was discharged home on oral linezolid (600 mg orally every 12 h) and levofloxacin (750 mg orally daily).

On presentation, the patient was in shock with a mean arterial pressure of 54 mmHg and severe metabolic acidosis (pH = 6.9) with respiratory alkalosis. His anion gap was 37, with a serum bicarbonate level of 4 mmol/L and a markedly elevated serum lactic acid level of 21 mmol/L. The patient was afebrile. His white blood cell count (WBC) was 13,300 cells/uL. Severe sepsis protocol was initiated and he was switched to broad-spectrum antibiotic therapy with meropenem and vancomycin. Blood and urine cultures were taken. Computed tomography (CT) of the thorax, abdomen and pelvis were done and did not reveal an active source of infection. A leukocyte scan of the right knee was negative for active infection. Inflammatory markers including erythrocyte sedimentation rate (ESR) and procalcitonin were within normal limits, and C-reactive protein (CRP) level was significantly lower than during prior admissions.

The next day, the patient’s WBC count normalized, but he remained severely acidemic, requiring one session of hemodialysis and bicarbonate supplementation. Within 48 h of ICU admission, continuation of antibiotics and aggressive hydration, his blood pH improved to 7.38 despite persistent elevated lactic acid levels (20 mmol/liter). All cultures were negative. Given these findings and in the absence of any clinical signs of infection. Linezolid was considered the cause of the severe lactic acidosis and antibiotics were discontinued.

On day 4 of hospitalization, the patient developed pancytopenia with a WBC count reaching 2200 cells/uL, platelets count of 36,000/uL, and hemoglobin of 8.8 g/dL with a reticulocyte count of less than 0.3%. The patient’s serum lactic acid level normalized on day 7 of hospitalization. His pancytopenia progressively improved and normalized on day 9 with a reticulocyte count of 6.2%.

**FIGURE 1:**
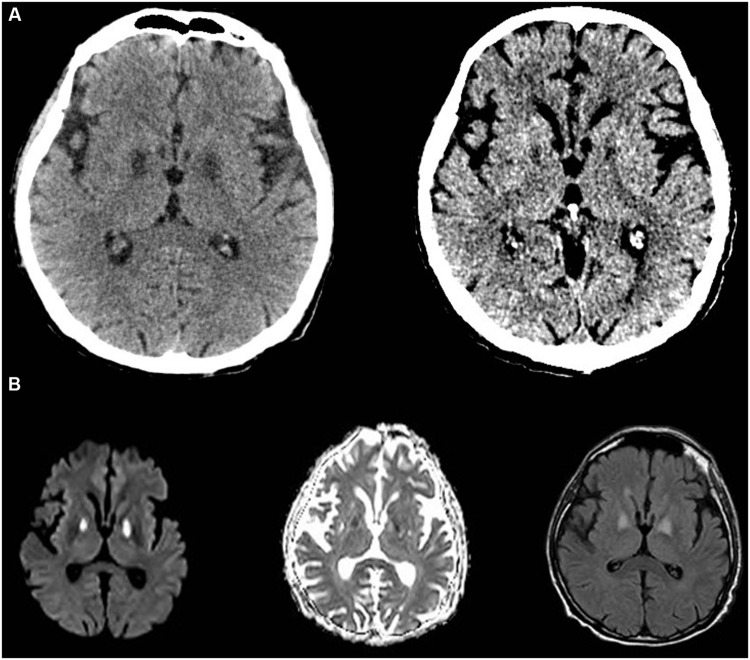
**Computed tomography-scan and MRI of the brain. (A)** Shows CT of the brain with bilateral globi pallidi hyper-intensities at day 5 (left) and 14 (right) from admission. **(B)** Shows the MRI images documenting acute ongoing stroke on day 6 on T1, T2, and flair captures, respectively.

The patient’s level of consciousness lagged behind; CT of the brain revealed unexpected hypodensities within the globi pallidi bilaterally (**Figure 1**). Magnetic resonance imaging [MRI and angiography (MRA) of the brain] demonstrated acute bilateral globus pallidus hyper-intensities consistent with necrosis (**Figure 1**). A lumbar puncture was done, and cerebrospinal fluid (CSF) analysis revealed normal lactate levels but elevated pyruvate levels 0.108 mmol/l (normal values between 0.006 and 0.019). Mental status improved slowly, but the patient remained weak with mainly proximal muscle weakness. A biopsy was taken from the right vastus lateralis muscle and showed widespread mitochondrial dysfunction with marked deficiency in cytochrome C oxidase, increased lipid globules within myofibers, neurogenic muscular atrophy, and increased regenerative activity. Further work up (acylcarnitine profile in blood and urine) was negative for other metabolic disorders.

Several weeks later, the patient’s symptoms improve drastically with rehabilitation.

## Background

Linezolid is a synthetic antibacterial of the oxazolidinone class. It is used to treat a variety of gram-positive infections, including methicillin-resistant *Staphylococcus aureus* (MRSA), vancomycin-resistant enterococci (VRE), *Nocardia* infections and multidrug-resistant tuberculosis. It has been lately encouraged as an alternative therapy in prosthetic infections (Morata et al., 2014). At times treatment duration is extended over several weeks. The first case of linezolid-induced lactic acidosis was described in 2003 (Apodaca and Rakita, 2003). Lactic acidosis occurs in 6.8% of patients receiving linezolid, and is particularly associated with longer durations of treatment (Im et al., 2015).

Linezolid acts by binding to the 23S ribosomal RNA of bacteria, thus inhibiting bacterial protein synthesis. It acts preferentially at the A-site of the ribosomal peptidyl transfer center to inhibit peptide bond formation (Leach et al., 2007; Wilson et al., 2008). The similarity between bacterial 23S ribosomal RNA and human mitochondrial 16S ribosomal RNA (Leach et al., 2007) was hypothesized to be the cause of human toxicity. In fact, genetic polymorphisms are speculated to be the main reason behind differential susceptibility to linezolid, and nucleotide polymorphism in the mitochondrial 16S ribosomal RNA in patients with linezolid-associated lactic acidosis has been reported (Palenzuela et al., 2005; Carson et al., 2007; Song et al., 2015)

Here we describe a patient with clinical and biochemical features secondary to linezolid toxicity secondary to a mitochondrial disorder.

## Methods

### Genetic and Enzymatic Studies

This study was approved by the American University of Beirut Institutional Review Board (Protocol Number: PBK11). One patient with linezolid-induced toxicity was recruited with written informed consent from the wife and children. Blood was taken for genetic analysis by dideoxy sequencing of previously reported haplogroup-defining regions. Spectrophotometric analysis of mitochondrial respiratory chain oxidative phosphorylation complexes was performed (El-Khoury et al., 2013) and compared to results of histochemical staining of muscle biopsy.

## Results

### Biomolecular Basis of Toxicity

**FIGURE 2:**
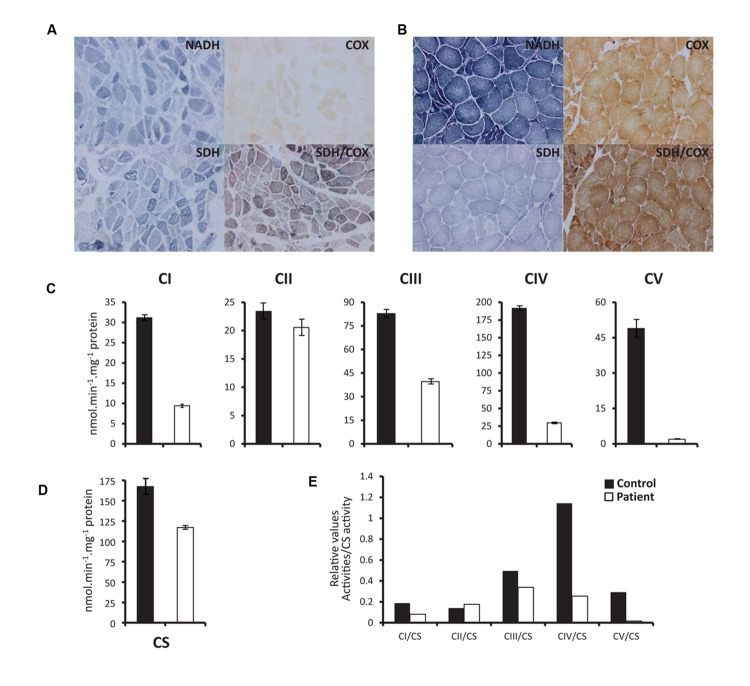
**Histochemical staining and spectrophotometric activity of respiratory chain enzymes. (A)** and **(B)** Show histopathologic stains of vastus lateralis muscle illustrating activities of NADH, COX (cytochrome oxidase), SDH, and COX/SDH in patient **(A)** and Control **(B)**. **(C)** Spectrophotometry measurements of specific activities of CI (NADH dehydrogenase), CII (Succinate dehydrogenase), CIII (Cytochrome bc1), CIV (cytochrome oxidase), and CV (ATP synthase) show marked drop of respiratory chain complexes activities in patient compared to control. **(D)** Citrate synthase (CS) specific activity measured as reference enzyme, which reflects the total mitochondrial mass. **(E)** Ratios of the absolute activities of the different complexes corrected to CS activity. Biochemical assessments were performed twice with the same control muscle biopsy used as a positive control. The patient’s enzymatic activities (nearly identical in both analyses) were far below the lower limit of our reference ranges.

The histochemical evaluation of the activity of all mitochondrial respiratory chain enzymes showed significant reduction of cytochrome oxidase (COX) enzymatic activity (**Figures 2A,B**). COX staining revealed a more severe loss of activity on histochemical stains than observed for other mitochondrial respiratory chain enzymes. This loss was also evident when the specific activity of each complex was assessed spectrophotometrically on patients’ muscle homogenate preparation. Indeed, we have shown that all mitochondrial respiratory chain complexes activities, but complex II (which is completely encoded from the nucleus), are depressed in the patient, with more severe loss in Complexes IV and V (**Figures 2C–E**).

### Gentic Basis of Toxicity

Sanger sequencing of the mitochondrial DNA was carried on our patient and the results were compared to available electronic databases as well as previously reported susceptibility loci. Our patient belongs to the J1 mtDNA haplogroup. This haplogroup is carried by 9.2% of non-hispanic whites in the United States (Mitchell et al., 2014) and 8–9% of the European population.

## Discussion

Linezolid toxicity is associated with 26% mortality (Im et al., 2015). The high incidence of death in patients with severe toxicity reflects such a systemic mitochondrial cytopathy with multi-organ dysfunction.

Linezolid interferes directly with mitochondrial protein synthesis and therefore with respiratory chain activity (Nagiec et al., 2005; Soriano et al., 2005). This results in severe clinical consequences such as lactic acidosis, myelosuppression, and peripheral and ocular neuropathies. This occurs due to severe mitochondrial dysfunction in highly active cells. The effect of linezolid on the neurologic system are not well-determined; a very small number of case reports showed the potential of linezolid to induce optic neuropathy, peripheral neuropathy, and encephalopathy (Ferry et al., 2005; Nagel et al., 2007; Narita et al., 2007; Fletcher et al., 2010). The previous reported cases of encephalopathy were transient, sometimes related to other coexisting sedating drugs (Ferry et al., 2005) and with negative brain MRI findings (Ferry et al., 2005; De Vriese et al., 2006; Fletcher et al., 2010). However, there are no prior reported cases of linezolid-induced stroke-like lesions in the globi pallidi bilaterally as in our patient. Stroke-like episodes are episodic events described previously with mitochondrial disorders – most frequently with MELAS syndrome (Goodfellow et al., 2012), Leigh’s encephalopathy and carbon monoxide or cyanide poisoning. Neurological manifestations occur late and are likely due to the weaker penetration to the brain tissue, while myelosuppression and myopathy tend to appear earlier. Most of the mitochondrial abnormalities normalize after linezolid withdrawal, with lactate normalization and clinical recovery as has occurred in this case and previously reported cases (Garrabou et al., 2007).

Various presentation of linezolid toxicity is secondary to varying ribosomal susceptibility. The mitochondrial haplogroup (J1 in our case) determines the cause, while the cumulative treatment duration dictates the spectrum. Certain mitochondrial haplogroups are more susceptible to linezolid effect than others (Pacheu-Grau et al., 2013); *in vitro*, the polymorphism decreases synthesis of mtDNA-encoded polypeptides in cells treated with linezolid. The J1 defining SNPs are close to the ribosomal peptidyl transferase center. This proximity and phenotype picture links the susceptibility hypothesis with molecular basis of toxicity. The prevalence of J1 haplogroup suggests that 8–9% of US and European patients are at risk of developing complications described above.

Patients present with severe lactic acidosis mimicking septic shock, develop myelosuppression, optic or peripheral neuropathies, and myopathies, and central nervous system disease as documented in this report–a picture suggestive of an acquired mitochondrial syndrome. Differentiating linezolid toxicity from septic shock and cerebrovascular events is essential in improving survival and patient outcome.

Hints such as bilateral globi pallidi lesions and abnormally high lactic acid levels (greater than 20 mmol/L) should not defer antibiotic therapy for other infective processes. However, careful choices of antibiotics in this population is required; vancomycin, for example, is also known to inhibit complex I of the respiratory chain (Arimura et al., 2012), and may result in additional mitochondrial dysfunction.

## Conclusion

Linezolid toxicity is associated with significant morbidity and mortality. It mimics septic shock delaying diagnosis. Up to 9% of Western population could be at risk. Bilateral globi pallidi necrosis presents in linezolid toxicity in the setting of systemic mitochondrial cytopathy. We recommend screening for J1 mitochondrial haplogroup in patients considered for prolonged therapy with the drug.

## Author Contributions

OA, MK, and PB conceived and wrote the research project, performed literature review, and wrote the manuscript. OA, RE, GN, and MA conducted experiments, performed analysis, and wrote manuscript. All authors read and approved the final manuscript.

## Conflict of Interest Statement

The authors declare that the research was conducted in the absence of any commercial or financial relationships that could be construed as a potential conflict of interest.
